# Pangenome-Wide Association Study in the *Chlamydiaceae* Family Reveals Key Evolutionary Aspects of Their Relationship with Their Hosts

**DOI:** 10.3390/ijms252312671

**Published:** 2024-11-26

**Authors:** Rosalba Salgado-Morales, Karla Barba-Xochipa, Fernando Martínez-Ocampo, Edgar Dantán-González, Armando Hernández-Mendoza, Manuel Quiterio-Trenado, Magdalena Rodríguez-Santiago, Abraham Rivera-Ramírez

**Affiliations:** 1 Laboratorio de Estudios Ecogenómicos, Centro de Investigación en Biotecnología, Universidad Autónoma del Estado de Morelos, Av. Universidad 1001, Col. Chamilpa, Cuernavaca CP 62210, Mexico; rosalba.salgadomo@docentes.uaem.edu.mx (R.S.-M.); edantan@uaem.mx (E.D.-G.); 2 Facultad de Ciencias de la Salud, Universidad Autónoma de Tlaxcala, Universidad 1, Tlaxcala de Xicohténcatl CP 90000, Mexico; karlamichellebarbaxochipa@gmail.com (K.B.-X.); magdalena482003@gmail.com (M.R.-S.); 3 Centro de Investigación en Dinámica Celular, Universidad Autónoma del Estado de Morelos, Av. Universidad 1001, Col. Chamilpa, Cuernavaca CP 62210, Mexico; fernando.martinez.phd@gmail.com (F.M.-O.); ahm@uaem.mx (A.H.-M.); 4 Programa de Estancias Posdoctorales por México 2022(3), Modalidad Académica-Inicial, Consejo Nacional de Humanidades, Ciencias y Tecnologías, Av. Insurgentes Sur 1582, Col. Crédito Constructor, Alcaldía Benito Juárez CP 03940, Mexico; 5 Centro de Investigación en Salud Poblacional, Instituto Nacional de Salud Pública, Av. Universidad 655, Cuernavaca CP 62100, Mexico; mquitero@insp.mx

**Keywords:** *Chlamydiaceae*, *Chlamydia*, *Chlamydophila*, pangenome, Pan-GWAS

## Abstract

The *Chlamydiaceae* are a family of obligate intracellular bacteria known for their unique biphasic developmental cycle. *Chlamydial* are associated with various host organisms, including humans, and have been proposed as emerging pathogens. Genomic studies have significantly enhanced our understanding of *chlamydial* biology, host adaptation, and evolutionary processes. In this study, we conducted a complete pangenome association analysis (pan-GWAS) using 101 genomes from the *Chlamydiaceae* family to identify differentially represented genes in *Chlamydia* and *Chlamydophila*, revealing their distinct evolutionary strategies for interacting with eukaryotic hosts. Our analysis identified 289 genes with differential abundance between the two clades: 129 showed a strong association with *Chlamydia* and 160 with *Chlamydophila*. Most genes in *Chlamydia* were related to the type III secretion system, while *Chlamydophila* genes corresponded to various functional categories, including translation, replication, transport, and metabolism. These findings suggest that *Chlamydia* has developed a high dependence on mammalian cells for replication, facilitated by a complex T3SS for intracellular manipulation. In contrast, the metabolic and functional diversity in *Chlamydophila* allows it to colonize a broad range of hosts, such as birds, reptiles, amphibians, and mammals, making it a less specialized clade.

## 1. Introduction

The *Chlamydiaceae* are an ancient family of obligate intracellular Gram-negative bacteria characterized by a unique biphasic developmental cycle [1,2]. This distinctive cycle alternates between extracellular infectious elementary bodies (EBs), which are metabolically slowed forms similar to spores, specialized for survival in the environment and for adhesion and entry into the host cell, and intracellular replicative reticulate bodies (RBs), which are the germinal forms that actively divide 8–10 times before redifferentiating into EBs [3,4,5]. These reside in a membrane-enclosed inclusion. After the replication of the RBs and the growth of the inclusion, they dedifferentiate into EBs, which are released from the host cells and continue the infectious process in other cells [6,7,8]. The life cycle of *chlamydia* is fundamental to the pathogenesis of these bacteria, as their survival depends on the complex interactions between the host and the pathogen to establish an intracellular niche, subvert the host’s cellular processes, acquire host-derived nutrients, and evade the host’s immune response [9,10,11].

The genomic organization in the *Chlamydiaceae* family is remarkably conserved and synteny, featuring compact and significantly reduced genomes with a length of approximately 1–1.2 Mbp and around 900 to 1200 coding sequences [12,13,14,15]. In most described species of the *Chlamydiaceae* family, the presence of a highly conserved *chlamydial* plasmid of approximately 6–8 kbp has been reported [16,17,18]. The compact genome observed in *Chlamydiaceae* species is a result of genomic streamlining rather than degradation, believed to arise from their adaptation to an intracellular lifestyle and co-evolution with their eukaryotic hosts [19,20,21,22].

The taxonomy of *Chlamydia* has been extensively revised in recent years, driven by genomic studies of this microorganism. In 1997, Everett and colleagues proposed, based on ribosomal RNA (rRNA) gene sequence analysis, dividing the *Chlamydiaceae* family into two genera, *Chlamydia* and *Chlamydophila* [23]. This led to the identification of nine species: *C. trachomatis*, *Chlamydia muridarum*, and *Chlamydia suis*, as well as *Chlamydophila* (Cp.) *abortus*, Cp. *caviae*, Cp. *felis*, Cp. *pecorum*, Cp. *pneumoniae*, and Cp. *psittaci*. Subsequently, Sachse et al., 2015 proposed the reclassification of the 11 species recognized at that time within the *Chlamydiaceae* family into a single genus, the genus *Chlamydia*. They concluded that neither the commonly used 16S rRNA sequence identity cut-off values nor parameters based on genomic similarity consistently separate the two genera. Notably, there is no easily recognizable phenotype, such as a host preference or tissue tropism, that would support a subdivision [24]. This debate continues to evolve as more genomic and phylogenetic studies provide new insights into these bacteria. In this study, we conducted a pangenomic and phylogenomic analysis of 101 genomes from the *Chlamydiaceae* family, ensuring complete representation of the currently recognized species. We applied a full pangenome association analysis to investigate genetic diversity differences between the two clades and the species they comprise to gain fundamental insights into chlamydial biology, host adaptation, and evolution.

## 2. Results

### 2.1. Architecture, Organization, and Functionality of the Pangenome of the Family Chlamydiaceae

**Figure 1:**
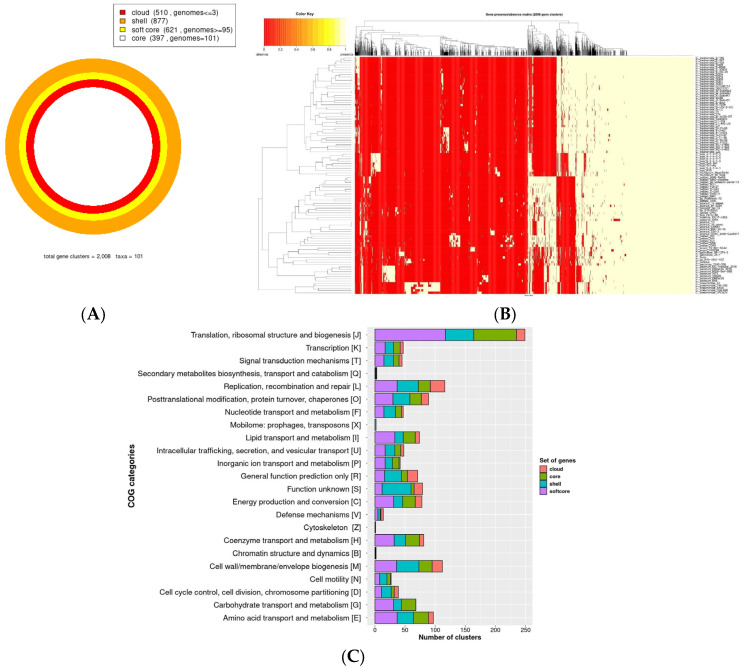
Overview and functional classification of the pangenome of the *Chlamydiaceae* family. (**A**) Partitioning of the pangenome with OMCL into the categories of core, soft-core, shell, and cloud, along with the number of gene clusters associated with each. (**B**) Distribution of gene clusters among the 101 genomes used in the analysis: yellow indicates presence and red indicates absence. (**C**) Distribution of COG functional categories in the pangenome of the *Chlamydiaceae* family is color-coded as follows: core (green), soft-core (purple), shell (blue), and cloud (coral). The number of proteins is shown on the *x*-axis, and the COG categories are displayed on the *y*-axis.

The pangenome analysis, which included 101 genomes from members of the *Chlamydiaceae* family, revealed a total pangenome size of 2 008 gene clusters, which were categorized into four occupancy classes. The largest category is the shell (remaining moderately conserved genes present in several genomes), comprising 877 gene clusters; the second category is the soft-core, containing 621 gene clusters (present in 95% of genomes); followed by the cloud, with 510 gene clusters (present in ≤2 genomes); and the smallest category is the core, consisting of 397 gene clusters (Figure 1A). The distribution of the 2008 gene clusters per genome can be seen in Figure 1B. Metabolic capabilities were evaluated through the functional annotation of the coding sequences (CDSs) in the pangenome of the *Chlamydiaceae* family, using the COG (Clusters of Orthologous Groups) database. The most enriched categories correspond to the Translation, ribosomal structure, and biogenesis [J]; Replication, recombination, and repair [L]; Cell wall/membrane/envelope biogenesis [M]; Amino acid transport and metabolism [E] and Posttranslational modification, protein turnover, chaperones [O] (Figure 1C).

### 2.2. Clustering Genomes into Groups That Share Similar Gene Sets

**Figure 2:**
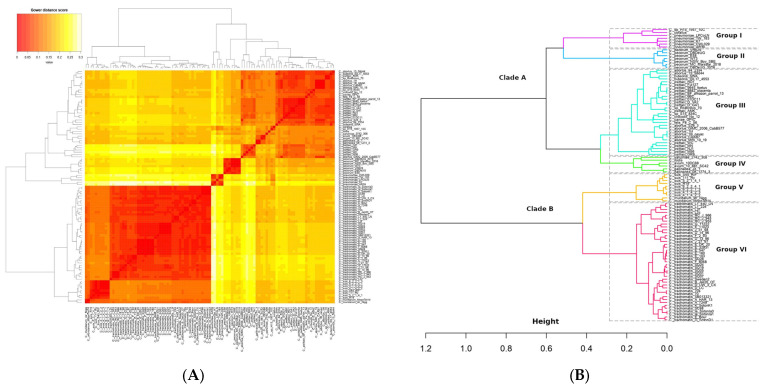
Pangenome clustering of the *Chlamydiaceae* family. (**A**) Heatmap clustering according to 2009 pangenome genes from 101 genomes of the *Chlamydiaceae* family. Gower distance score based on presence—absence pangenome matrix: Red = more similar, white = less similar. (**B**) Hierarchical clustering dendrogram based on the Gower dissimilarity matrix applying Average Silhouette Width statistics for groups generation based on genetic diversity. The groups are delineated by the dashed lines.

To gain a broader understanding of the genetic diversity contained in the pangenome, as well as the differences and similarities between various species, we applied Gower’s distance analysis to the pangenomic matrix. The heatmap shows distinct clusters, indicating groups of genomes that share more genes (low Gower distance or values closer to 0) and others that are more divergent (high Gower distance or values closer to 1). The analysis reveals the formation of two major clades, A and B, corresponding to the genera *Chlamydophila and Chlamydia*, respectively. The clade A corresponding to the *Chlamydophila* genus, various species such as *C. abortus*, *C. buteonis*, *C. psittaci*, *C. crocodili*, *C. caviae*, *C. felis*, *C. corallus*, *C. sanziniae*, *C. pneumoniae*, *C. pecorum*, *C. gallinacea*, *C. avium*, and *C. ibidis* are grouped together, showing higher dissimilarity values and indicating a highly heterogeneous and divergent clade. The clade B corresponding to *Chlamydia* includes only the species *Chlamydia trachomatis*, *Chlamydia muridarum*, and *Chlamydia suis*, which exhibit low dissimilarity values, indicating highly similar genomes and reduced genetic diversity. This suggests that the functions and processes represented in clade B may be conserved among these species, possibly reflecting similar genetic adaptations or an evolutionary proximity between them as shown in Figure 2A. Certain species, such as *Chlamydia trachomatis*, *C. psittaci*, and *C. pecorum*, form tight subclusters, showing a high genetic similarity within the same species but a greater distance from others (Figure 2A).

We then applied hierarchical clustering analysis to identify species groups with a statistically consistent genetic similarity, based on the matrix converted to Gower’s distance. This analysis indicated the presence of six groups with different genetic diversity, four groups found within the *Chlamydophila* genus and two present in *Chlamydia* as shown in Figure 2B. Group I consists of *C. pneumoniae*, *C. corallus*, and *C.* sp. *H15-1957-10C* (serpentis); Group II is composed solely of *C. pecorum*; Group III is the most diverse, including *C. abortus*, *C. buteonis*, *C. psittaci*, *C. crocodili*, *C. caviae*, and *C. felis*. Group IV includes *C. sanziniae*, *C. ibidis*, *C. avium*, and *C. gallinaceae*; Group V consists of *C. muridarum* and *C. suis*; and Group VI is made up solely of the *C. trachomatis* species (Figure 2B).

### 2.3. Pan-GWAS Analysis Reveals Unique Genes in Chlamydia and Chlamydophila

**Figure 3:**
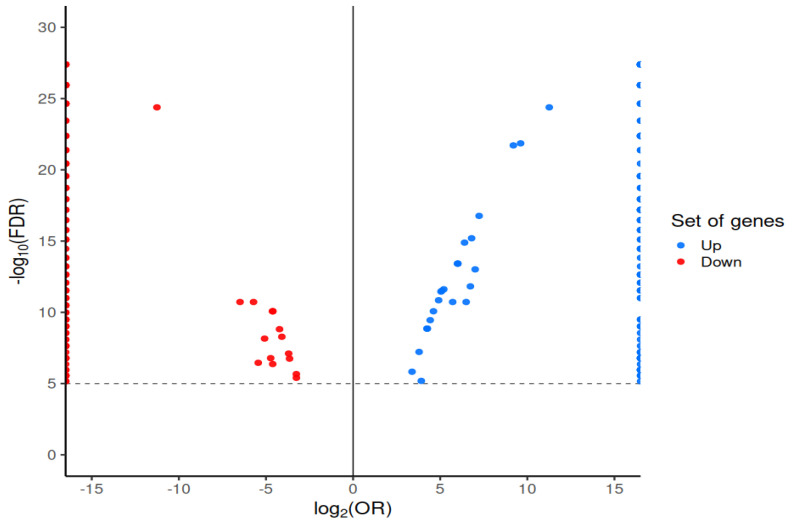
Pan-GWAS analysis to identify unique genes associated with the genera *Chlamydia* and *Chlamydophila*. Volcano plots illustrate the relationship between statistical significance (−log10) and effect size, represented as the log-transformed (base 2) odds ratio, for the assignment of unique genes in the genera *Chlamydia* and *Chlamydophila*. The horizontal dash line indicates the statistical significance threshold for pan-WGAS, which is determined using the Bonferroni adjustment.

The presence of unique gene clusters associated with *Chlamydia* and *Chlamydophila* was investigated through a Pan-GWAS analysis conducted using the phylogeny-based Scoary method. The analysis revealed 289 genes differentially abundant between both, surpassing the adjusted *p*-value threshold of 1 × 10⁻^5^ as shown in Figure 3. The COG annotation showed that a total of 19% of the genes were classified as having an unknown function, 9% as related to translation, ribosomal structure, and biogenesis, and 7.5% as involved in cell wall/membrane/envelope biogenesis, 7.5% as inorganic ion transport and metabolism, 5.88% as replication, recombination, and repair, and 5.34% as intracellular trafficking, secretion, and vesicular transport.

The main abundant genes (with the highest statistical significance and present exclusively in this genus) in *Chlamydia* were the genes incE and incF, crpA, gspE, scc2, copD, sohB, murE, and recB; most are related to cell invasion and survival inside host cells. In contrast, some of the less abundant genes were groL2, adA, sigD, artM, uvrC, tolB, Int, rapA, and ytlF. In *Chlamydophila*, the most abundant genes included: *groEL-2*, euo, oppF_2, sdhA, and *bioB*, encodes for a biotin synthase, that is involved in the final stage of the biotin biosynthesis pathway, an essential compound that acts as a cofactor for several enzymes in cellular metabolism, priA, cutA, epsE, mrdA, and sdhC; many of them belong to the central metabolism, as shown in Table 1.

As part of the analysis, we set out to identify virulence factors related to the unique genes detected in *Chlamydia* and *Chlamydophila*. This analysis showed that *Chlamydia* possesses a higher number of elements in its genomes related to the type III secretion system, such as *cdsD*, *cdsG*, *copD*, *scc2*, and *scc3*, as well as its effectors, including the genes *inaC*, *incB*, *incD*, *IncE*, *IncG*, and *tepP*, compared to genomes belonging to *Chlamydophila*, which only contain *cdsD*, *copD*, and *scc2*, along with the effectors *ChlaOTU*, *incA*, and *tarp*, respectively. We also identified a greater number of polymorphic membrane protein (PMP) genes in *Chlamydia*, detecting pmpA, pmpC, pmpD, pmpG, and pmpH, whereas in *Chlamydophila* we only detected pmp2A and pmp11G. All these elements are strongly linked to pathogenicity mechanisms and host specificity.

### 2.4. Metabolic and Functional Diversity of the Accessory Genome in the Groups of Species That Comprise the Genera Chlamydia and Chlamydophila

To gain a more comprehensive understanding of the genetic and functional diversity within different groups of species of *Chlamydia* and *Chlamydophila*, and to gather insights into the differences related to their host interaction strategies, as well as their evolutionary and ecological adaptations, we proceeded to analyze the accessory genome in each of these groups. These genes are typically not essential for the survival of the organism under all conditions but may confer advantages in specific environments.

**Figure 4:**
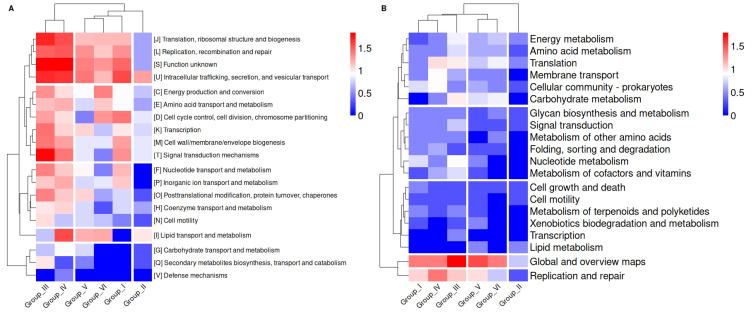
Heatmaps and hierarchical clustering of differentially abundant COG and KEGG categories across the different groups. The functions are divided according to COG and KEGG level 2 categories with logarithm base 10 normalized values. (**A**) Functional classification of protein-coding genes present in the accessory genome of the different groups based on the abundance of clusters of orthologous groups of proteins (COGs) (**B**) Functional classification of protein-coding genes present in the accessory genome of the different groups based on the abundance of KEGG level 2. The color code represents the level of abundance.

The pattern observed in both heatmaps reflects functional adaptations related to the intracellular lifestyle of the *Chlamydiaceae* family, with categories related to translation, replication, and membrane transport being the most represented across all groups. This suggests that these processes are essential for survival and proliferation within their hosts. Additionally, the under-representation of categories such as energy metabolism and secondary metabolism highlights the strong dependence of *Chlamydiaceae* members on the intracellular environment to meet their basic metabolic needs as shown in Figure 4A,B.

The analysis showed that groups I, III, and IV belonging to *Chlamydophila* exhibit a very similar functional profile in their accessory genomes, highlighting a very high abundance in various categories such as translation (J), DNA replication and repair (L), and membrane transport (KEGG: Membrane Transport), as well as elements related to metabolism. The abundance in these categories indicates that these groups rely heavily on the translation and replication machinery, which is essential for proliferation within host cells, but they also demonstrate significant metabolic versatility. This suggests that members of these groups may be better adapted to rapid intracellular replication, efficiently utilizing host resources for these processes compared to other groups.

In both heatmaps, Group II stands out due to the under-representation of several functional categories, which could indicate a higher specialization of species in this group for specific intracellular niches. This would allow them to depend more on the host for most of their metabolic and biosynthetic functions. Rather than replicating as actively as other groups, they might be more adapted to persistence or latency strategies within host cells (Figure 4A,B). Groups V and VI from *Chlamydia* exhibited moderate to low representation in most functional categories compared to the other groups. The most represented category in both groups is lipid transport and metabolism (I). Some key differences between the two groups are that Group V shows categories related to post-translational modifications and transcription as highly represented, while in Group VI, the most represented categories are related to energy production and conversion (C), cell cycle control (D), and amino acid metabolism and transport (E). These differences between Group V and VI may reflect varying degrees of intracellular specialization. Group VI appears more specialized in an environment where a high replication rate or biosynthetic activity is not required, whereas Group V shows more signs of a more active replicative strategy (Figure 4A,B).

### 2.5. Phylogenomic Analysis of the Chlamydiaceae Family

To compare and understand the evolutionary relationships between the different species groups generated from the pangenome, considering both the genes shared by all genomes as well as the variable genes present in only some species, we performed a phylogenomic analysis using 383 single-copy genes from the core genome.

**Figure 5:**
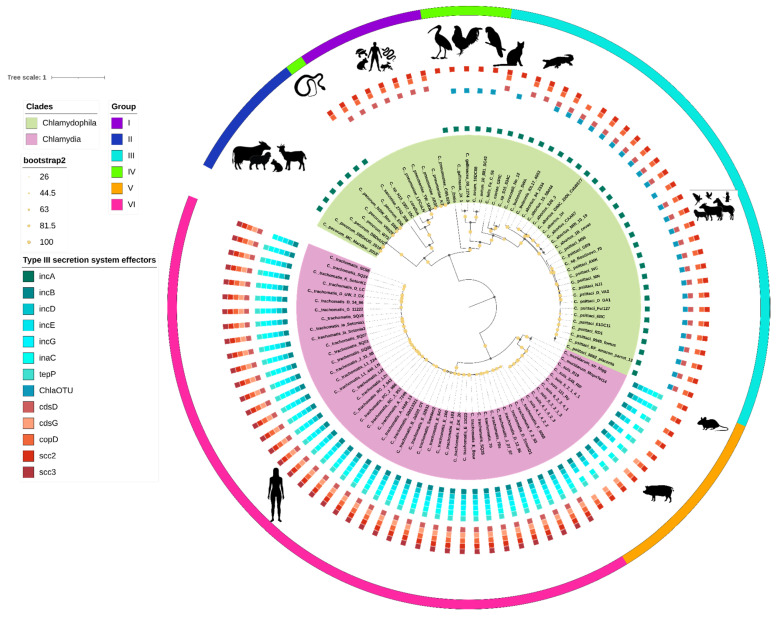
Best maximum-likelihood core-genome phylogeny for the *Chlamydiaceae* family. The ML tree is based on 79 top-scoring markers defined by GET_PHYLOMARKERS, selected for their optimal phylogenetic attributes. Nodal support values are color-coded as shown in the legend and correspond to ultrafast bootstrap (UFBoot2) values support. The scale represents the number of expected substitutions per site under the best-fit GTR2 + F + R3 model. The phylogeny corresponds to the tree with the highest score (lnL = −806,112.377) found among 10 independent searches in IQTREE.

To determine and quantify the topological correlation between the phylogeny of the core genome and the dendrogram displaying the genetic diversity within different groups, we measured the congruence using the normalized Robinson–Foulds index (nRF). An nRF score of 0 or close to it indicates that the trees under investigation show a high level of congruence, while scores close to or equal to 1 indicate a lack of congruence. Our analysis showed that the two trees exhibit high similarity with an nRF value of 0.412 (*t*-test *p* value < 0.001), indicating a close relationship between the core phylogeny and the full gene content (see Figure S1). The structure of the groups remained consistent across both trees, with the exception of *C. sanziniae*, a member of Group IV, which in the core genome phylogeny was grouped within Group I, alongside *C. pneumoniae*, *C. corallus*, and *C. sp. H15_1957_10C* (serpentis). This suggests that there might be certain interspecies similarities in the gene repertoire of *C. sanziniae* and species corresponding to group IV. However, such similarities do not prevent lineage-specific segregation as shown in Figure 5.

We also conducted a search for the hosts in which these species have been reported and isolated. What we can observe is that members of the genus *Chlamydophila* have a very wide host diversity, ranging from birds, amphibians, and reptiles to mammals. In contrast, members of the genus *Chlamydia* have only been reported in mammals (humans, pigs, and mice). This could be related to the fact that members of the genus *Chlamydia* possess a greater number of effectors and structural components related to the type III secretion system (T3SS), as detected in our analysis. The quantity and diversity of T3SS components in the genome of pathogens are closely related to their ability to adapt to and specialize in different hosts and cellular niches, making it a key factor in their evolution. In contrast, members of the genus *Chlamydophila* exhibit greater diversity in components related to replication, translation, and general metabolism, which has allowed them greater versatility in colonizing a wide range of hosts (Figure 5).

## 3. Discussion

In this analysis, we included all available genomes with the highest assembly quality, while also ensuring representation across the entire *Chlamydiaceae* family, comprising 19 currently recognized species [25]. Our results reveal an open pangenome for the *Chlamydiaceae* family, characterized by the shell and cloud gene subsets being the largest, which is a feature of open pangenomes. This is supported by the rarefaction curve obtained in our analysis (see Figure S2), which shows an ill-defined asymptote. The dynamic nature of the pangenome plays a crucial role in bacterial evolution, including niche adaptation, inter- and intra-species competition, pathogenic mechanisms, and antimicrobial resistance. Despite the restricted intracellular niche in the *Chlamydiaceae* family, research such as that by Gomes et al. (2007) demonstrated a high rate of recombination in *C. trachomatis* at very specific points in the genome [26]. The most compelling evidence of horizontal gene transfer processes among these organisms was provided by Marti et al. (2022) in co-culture studies. They used *C. suis*, which carried an incorporated genomic segment corresponding to the *tetC* gene. In the co-culture, *C. trachomatis* and *C. muridarum*, but not *C. caviae*, were able to integrate the resistance gene into their genomes. Genomic regions with evidence of recombination include the IncA genes, pmps, tarp, etc. [27]. These types of findings could help explain the pangenomic characteristics observed in the *Chlamydiaceae* family. These findings are consistent with previous analyses. Sigalova et al. 2019 reported that the pan-genome of the *Chlamydia* genus is formally open, with a total size of 2909 genes, and suggested that most of the genetic gains are due to paralogization and sequence divergence of some commonly present genes and paralogous gene families, including Pmps, PLDs, and Incs [28]. Similarly, Dharamshi et al. (2023) analyzed how gene gain processes have significantly impacted the evolutionary trajectory of the *Chlamydiae* phylum, increasing metabolic complexity in some *chlamydia* families that infect protists [29]. In general, gene acquisition in the *Chlamydiaceae* family appears to be restricted to very specific functions related to its intracellular lifestyle, as reflected in the functional composition of both the core and accessory genomes, where the most represented functions are associated with processes like translation, ribosomal structure and biogenesis, replication, recombination and repair of DNA. This suggests that protein synthesis processes are highly active, which is consistent with the fact that members of the *Chlamydiaceae* family are obligate intracellular bacteria that rely on a high translation rate to reproduce and replicate their DNA quickly, in order to propagate the infection once inside the host cell. Similarly, the low number of functional elements related to macromolecule biosynthesis and secondary metabolite pathways is consistent with what is known about the *Chlamydiaceae* family; as an obligate intracellular parasite, it heavily relies on the host’s resources to meet many of its metabolic needs [30,31].

The hierarchical clustering of the pangenomic matrix and the delineation of groups with statistically consistent genetic diversity revealed the formation of two major clades within the *Chlamydiaceae* family, corresponding to the genera *Chlamydophila* and *Chlamydia*. This clustering was observed not only with the pangenomic matrix but also when we performed a cgANIb analysis and with the core genome phylogeny, indicating two consistent groups that have followed divergent evolutionary paths with different strategies for host colonization and interaction (see Figures S3 and S4). This phylogenomic analysis supports the idea of two completely distinct clades. Our data could help partially resolve the taxonomic issues within the *Chlamydiaceae* family, as Sachse et al. (2015) concluded that neither the commonly used 16S rRNA sequence identity cut-off values nor parameters based on genomic similarity consistently separate the two genera [24].

Our analysis revealed a total of 289 differentially abundant genes between the clades, with 129 found exclusively in the genus *Chlamydia* and 160 in *Chlamydophila*. The primary differences lie in the content of genetic elements related to the T3SS, Inc inclusion proteins, and polymorphic membrane proteins (pmp), all of which are closely associated with adhesion, pathogenicity, and virulence mechanisms in these bacteria [32]. These elements are more abundant in the genus *Chlamydia*. There is a relationship between the quantity and functionality of T3SS elements and host adaptation and specificity, as these elements modulate various cellular functions, such as apoptosis inhibition, immune response evasion, and intracellular trafficking manipulation, facilitating bacterial replication and survival. Conversely, most of the genes detected in *Chlamydophila* and in the groups within that clade showed broader functional diversity (genetic elements related to metabolism) and a considerable decrease in elements related to pathogenicity and virulence. In contrast to other obligate intracellular and human pathogenic bacteria, such as *Rickettsia* and *Anaplasma*, the only systems detected were those corresponding to type I and IV secretion systems, which play a role in infection processes, the replicative cycle, and host immunity [33]. However, obligate intracellular pathogens of the *Chlamydiaceae* family possess a highly evolutionarily conserved non-flagellar T3SS, which is an absolute requirement for their survival and propagation within the host, with about one-seventh of the genome dedicated to genes associated with the T3SS apparatus, chaperones, and effectors. This is indicative of the different evolutionary strategies that intracellular pathogens have followed to colonize their hosts [34]. These findings could have a significant impact on clinical aspects such as the detection and treatment of *chlamydia*, as many of its members are considered to be potentially zoonotic. According to the U.S. Centers for Disease Control and Prevention (CDC), they are classified as Category B biological agents, meaning they are considered moderately easy to spread, may result in moderate morbidity rates and low mortality rates, and require specific enhancements in diagnostic capacity and increased disease surveillance [35].

Similarly, we detected considerable genetic diversity within the *Chlamydiaceae* family. The clade corresponding to *Chlamydophila* showed the greatest genetic diversity according to the calculated Gower distances, defining four species groups. Groups I, III, and IV exhibited the highest diversity of gene elements related to replication, translation, as well as energy metabolism and the metabolism of amino acids and nucleotides. This diversity positively correlates with the number of hosts they infect. Members of *Chlamydophila*, with greater metabolic and functional versatility, colonize a wide variety of hosts, including reptiles, amphibians, birds, and mammals. These observations are consistent with reports on environmental *chlamydias*, where a highly dynamic accessory genome and expanded metabolic potential have been observed [29,31]. The species comprising Groups I, III, and IV have been primarily isolated from farm animals (sheep, cattle, horses), cats, wild birds, snakes, amphibians, koalas, and some rodents. However, we also observed within the same clade Group II, which was the least diverse, consisting solely of *C. pecorum*, which has been reported in farm animals [36,37,38,39,40,41]. This could suggest a wide variety of strategies employed in colonization and replication levels within their hosts. On the other hand, Groups V and VI, corresponding to the *Chlamydia* clade, showed moderate diversity with some distinct functional categories, such as lipid transport and metabolism, and a very high abundance of elements related to the T3SS. Surprisingly, members of Group V (*C. suis* and *C. muridarum*) and Group VI (*C. trachomatis*) have only been detected in mammals such as pigs, rodents, and humans [42,43,44]. Therefore, the specificity and adaptation to a single group of animals could be determined by the abundance of T3SS-related genes linked to pathogenicity and virulence in *Chlamydia*. This suggests that these genes play a crucial role in how these organisms interact with their host. Zoonotic transmission of *C. suis* species to humans has also been documented [45,46], so this species, along with *C. muridarum* due to their high genetic similarity, could represent potential human pathogens. This study provides new insights into the molecular ecology, genomic diversity, and evolution of the *Chlamydiaceae* family.

## 4. Materials and Methods

### 4.1. Genomic Datasets

The publicly available complete genome sequences in the NCBI datasets for the *Chlamydiaceae* were downloaded in September 2023. A total of 101 genomes were downloaded from the RefSeq database discarding those without annotation and atypical genomes and ensuring full representation of the *Chlamydiaceae* family (Table S1). We used CheckM v2.0.1 quality analysis to preserve only high-quality genomes with parameters such as >90% completeness and <5% contamination [47].

### 4.2. Calculation of Core and Pangenome of the Genus Chlamydia

All genomes were re-annotated using Prokka v1.14.6 [48]. These annotated assemblies were then entered into GET_HOMOLOGUES v3.7.1 [49,50]. Ortholog clusters were created using the collection of scripts written in Perl in GET_HOMOLOGUES applying three different algorithms Bidirectional best hit (BDBH) [51], Clusters of Orthologous Genes (COG triangles) [52] and Ortho Markov Cluster (OMCL, inflation value 1.5) [53] for the identification and clustering of CDS in ortholog clusters with the following parameters: -E (E-value) < 1 × 10^−5^ for blastp searches, -C (coverage in BLAST pairwise alignments) 75% minimum alignment coverage, and -D Pfam domain search and composition when defining similarity-based orthology [54].

The core genome defined as the orthologous clusters present in all genomes was determined by the compare_cluster.pl script using the consensus of the three algorithms with the option -t 101 (corresponding to the total number of genomes). For the case of the pangenome, the same script was used; however, this was determined only with the OMCL and COGtriangles algorithms with the following options -t 0 (reporting all pangenome clusters), -m producing the pangenome intersection matrix and -T producing the pangenome tree based on parsimony. The remaining subsets of the pangenome—softcore (present in 95% of genomes), shell (>2 genomes but <95% of genomes) and the cloud (<=2 genomes)—were determined using the parse_pangenome_matrix.pl script and the -s option reporting the above clusters.

### 4.3. Pan-GWAS Analysis for the Identification of Unique Genes

For the Pan-GWAS analysis, we used Scoary v1.6.16 [55], which is based on a phylogenetic approach and calculates associations between all genes and accessory genome traits to identify unique genes related to different clades and groups at the pangenomic level, with a *p* value of 1 × 10^−5^. To control for false positives due to multiple comparisons, the Benjamini–Hochberg method was used to adjust the *p* values and determine the threshold for the adjusted *p* value.

### 4.4. Phylogenomic Analysis

Single-copy orthologous genes were identified using the three search and clustering algorithms in GET_HOMOLOGUES v3.7.1. The consensus of orthologous genes from the three algorithms was used for the phylogenetic reconstruction of the *Chlamydiaceae* family using the GET_PHYLOMARKERS v2.0.1 pipeline [56]. The former were first aligned with clustal-omega [57] and then used by pal2nal [58] to generate codon alignments. These were subsequently scanned with the Phi-test [59] to identify and discard those with significant evidence for recombinant sequences. Maximum-likelihood phylogenies were inferred for each of the non-recombinant alignments using IQ-TREE v.2.3.1 [60] by default, which performs model selection with ModelFinder [61]. The resulting gene trees were screened to detect “outliers” with help of the R package kdetrees v.0.1.5 [62]. In a third step, the phylogenetic signal of each gene-tree is computed based on mean branch support values [63], keeping only those above a user-defined mean Shimodaira-Hasegawa-like (SH-alrt) bipartition support [64] threshold (“-m 0.75” by default). The set of alignments passing all filters are concatenated and subjected to maximum-likelihood (ML) tree searching, using by default IQT with model fitting, to estimate the genomic species-tree.

### 4.5. Hierarchical Clustering

From the binary pangenome matrix generated with get_homologues.pl and compare_clusters.pl using the options -t 0 -m, it was converted to a Gower distance matrix using the script hcluster_pangenome_matrix.sh, which utilizes the hclust and heatmap.2 functions in R. Once obtained, the ward.D2 clustering algorithm was applied using the silhouette-width statistic to evaluate the clustering quality, with n = 1000 bootstrap replicates, S = 50 independent searches, and a maximum number of clusters *k* = 15.

### 4.6. Functional Annotation of Genes

For the functional classification of pangenomic orthologous clusters, information from the Pfam, GenBank, Uniprot, and KEGG databases was used, utilizing the accession numbers identified by GET_HOMOLOGUES, as well as the protein sequences of the orthologs, which were processed using the EggNOG v5.0.2 [65,66] and KofamScan v1.3.0 software [67].

## Figures and Tables

**Table 1:** Top 10 ranked genes identified in *Chlamydia* and *Chlamydophila* by the pan-genome-wide association analysis.

Gene	*p* Value	Genus	^a^ COG Category	Annotation
*incE*	5.00 × 10^−30^	*Chlamydia*	M	Inclusion membrane protein
*incF*	5.00 × 10^−30^	*Chlamydia*	M	Inclusion membrane protein
*crpA*	5.00 × 10^−30^	*Chlamydia*	M	Cysteine-rich outer membrane proteins
*gspE*	5.00 × 10^−30^	*Chlamydia*	M	Type II/IV secretion system protein
*scc2*	5.00 × 10^−30^	*Chlamydia*	-	Tetratricopeptide repeat
*copD*	5.00 × 10^−30^	*Chlamydia*	M	Secretion system effector C (SseC) like family
*sohB*	2.60 × 10^−28^	*Chlamydia*	OU	Peptidase family S49
*murE*	1.70 × 10^−24^	*Chlamydia*	-	UDP-N-acetylmuramoyl-L-alanyl-D-glutamate-2,6-diaminopimelate
*recB*	1.70 × 10^−24^	*Chlamydia*	L	Helicase nuclease
*mgtE*	1.91 × 10^−23^	*Chlamydia*	M	Magnesium transporter
*uvrA*	1.50 × 10^−21^	*Chlamydia*	L	Excinuclease ABC subunit A
*trpC*	7.39 × 10^−20^	*Chlamydia*	E	indole-3-glycerol-phosphate synthase
groEL_2	5.00 × 10^−30^	*Chlamydophila*	O	Chaperonin
Early upstream open	1.10 × 10^−20^	*Chlamydophila*	S	Belongs to the EUO family
oppF_2	4.51 × 10^−19^	*Chlamydophila*	-	Oligopeptide transport ATP-binding protein
sdhA	4.83 × 10^−7^	*Chlamydophila*	C	Succinate dehydrogenase
bioB	2.60 × 10^−28^	*Chlamydophila*	H	Biotin synthase
priA	2.60 × 10^−28^	*Chlamydophila*	-	Primosomal protein N’
cutA	2.60 × 10^−28^	*Chlamydophila*	P	Divalent-cation tolerance protein
epsE	2.60 × 10^−28^	*Chlamydophila*	U	General secretion pathway protein
mrdA	2.60 × 10^−28^	*Chlamydophila*	M	Penicillin-binding protein
sdhC	1.30 × 10^−26^	*Chlamydophila*	C	Succinate dehydrogenase

^a^ COG annotation category: C, energy production and conversion; E, Amino acid transport and metabolism; H, Coenzyme transport and metabolism; L, replication, recombination, and repair; M, cell wall, membrane, and envelope biogenesis; O, posttranslational modification, protein turnover, and chaperones; P, Inorganic ion transport and metabolism; U, Intracellular trafficking, secretion, and vesicular transport; S, function unknown.

## Data Availability

Data are contained within the article or Supplementary Material.

## Supplementary Materials

The following supporting information can be downloaded at: https://www.mdpi.com/article/10.3390/ijms252312671/s1.
