# Supplementary material for: Pangenome-Wide Association Study in the Chlamydiaceae Family Reveals Key Evolutionary Aspects of Their Relationship with Their Hosts
Source: Int J Mol Sci. 2024 Nov 26;25(23):12671. doi: 10.3390/ijms252312671 (PMC11641800; doi:10.3390/ijms252312671)
Supplement: Supplementary file 1 [file ijms-25-12671-s001.zip › Supplementary_files.pdf]

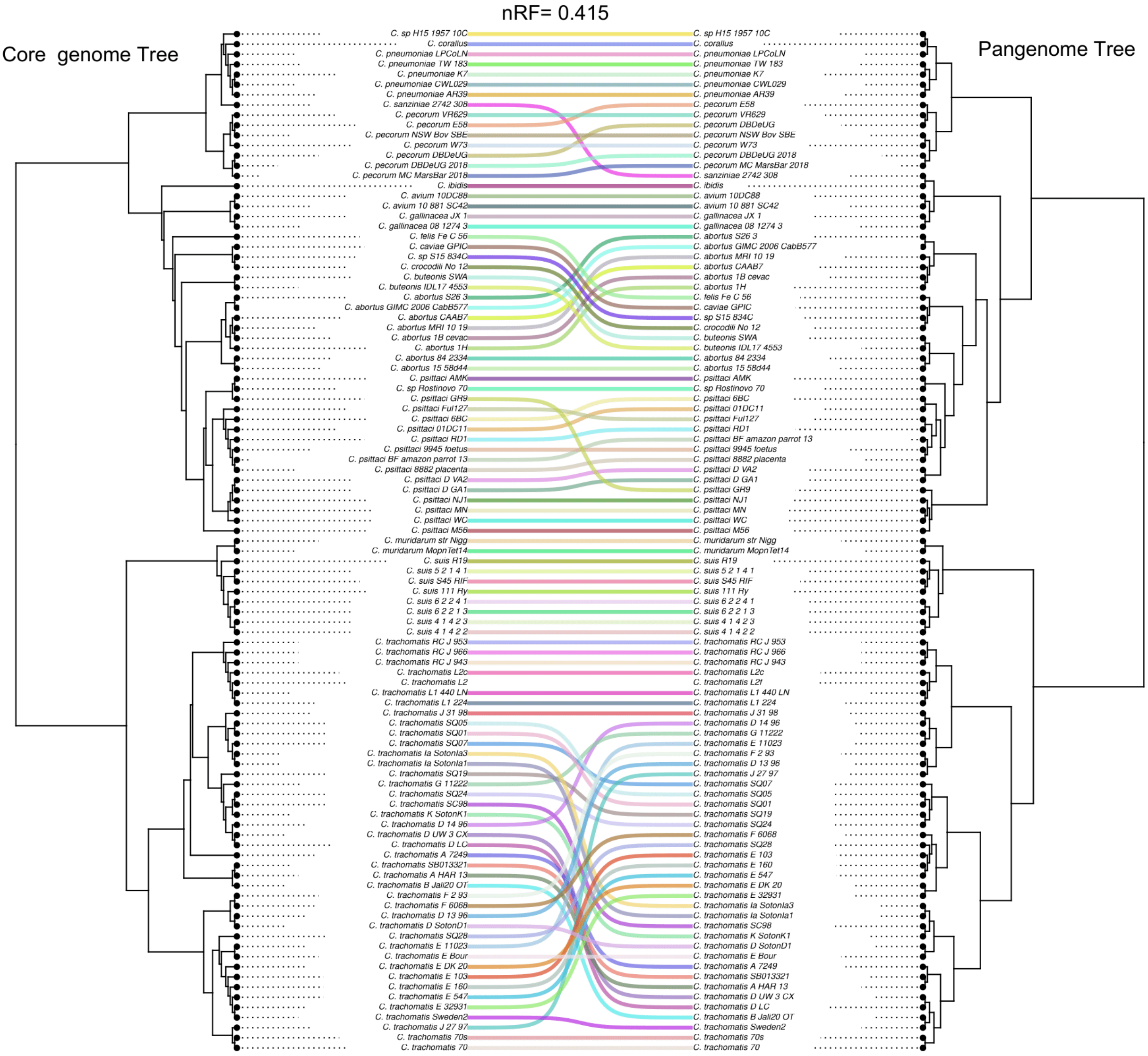

Figure S1. Comparison of two phylogenetic trees constructed using the core genome and pan-genome, respectively. (a) Phylogenetic tree based on the concatenated 79 top-scoring markers defined by GET_PHYLOMARKERS. (b) Pan-genome tree based on the presence or absence of the gene in the pan-genome. Normalized Robinson-Foulds (nRF) scores are used to measure the congruence between the two trees.

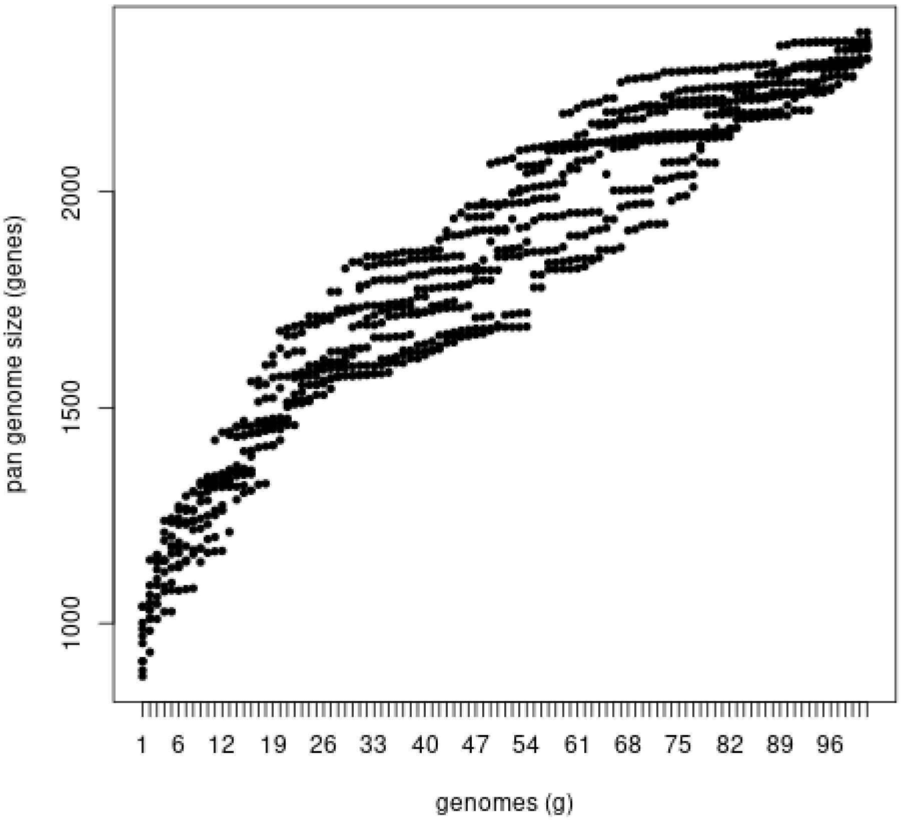

Figure S2. Pan-genome rarefaction curve of the Chlamydiaceae family. Rarefaction curve of the 101 genomes calculated from random combinations of strains. The iterations and combinations are shown as a cloud of points, indicating the total number of non-redundant genes included in the pan-genome as genomes are added to the analysis

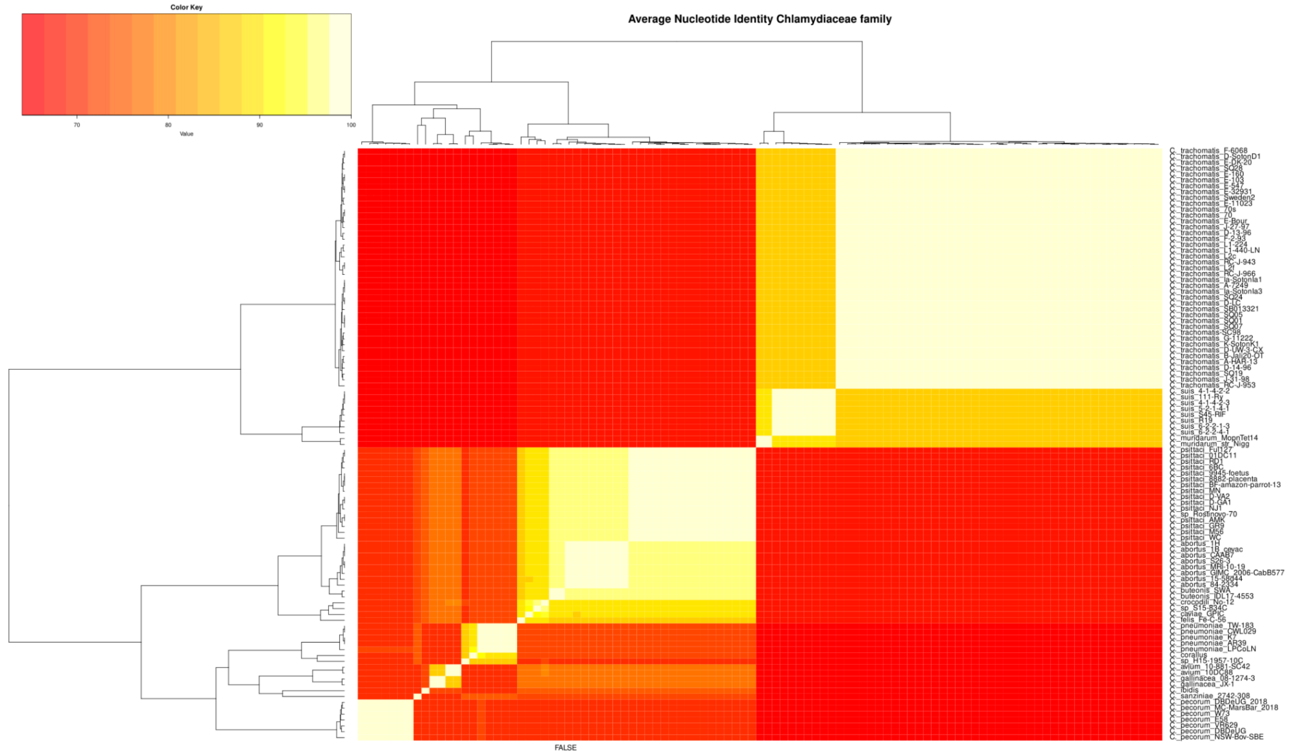

Figure S3. Average nucleotide identity (ANIb) heatmap. The heatmap was derived from an average nucleotide identity matrix determined from the high similarity (light yellow) and low similarity (red) of CDSs derived from the *Chlamydiaceae* genomes.

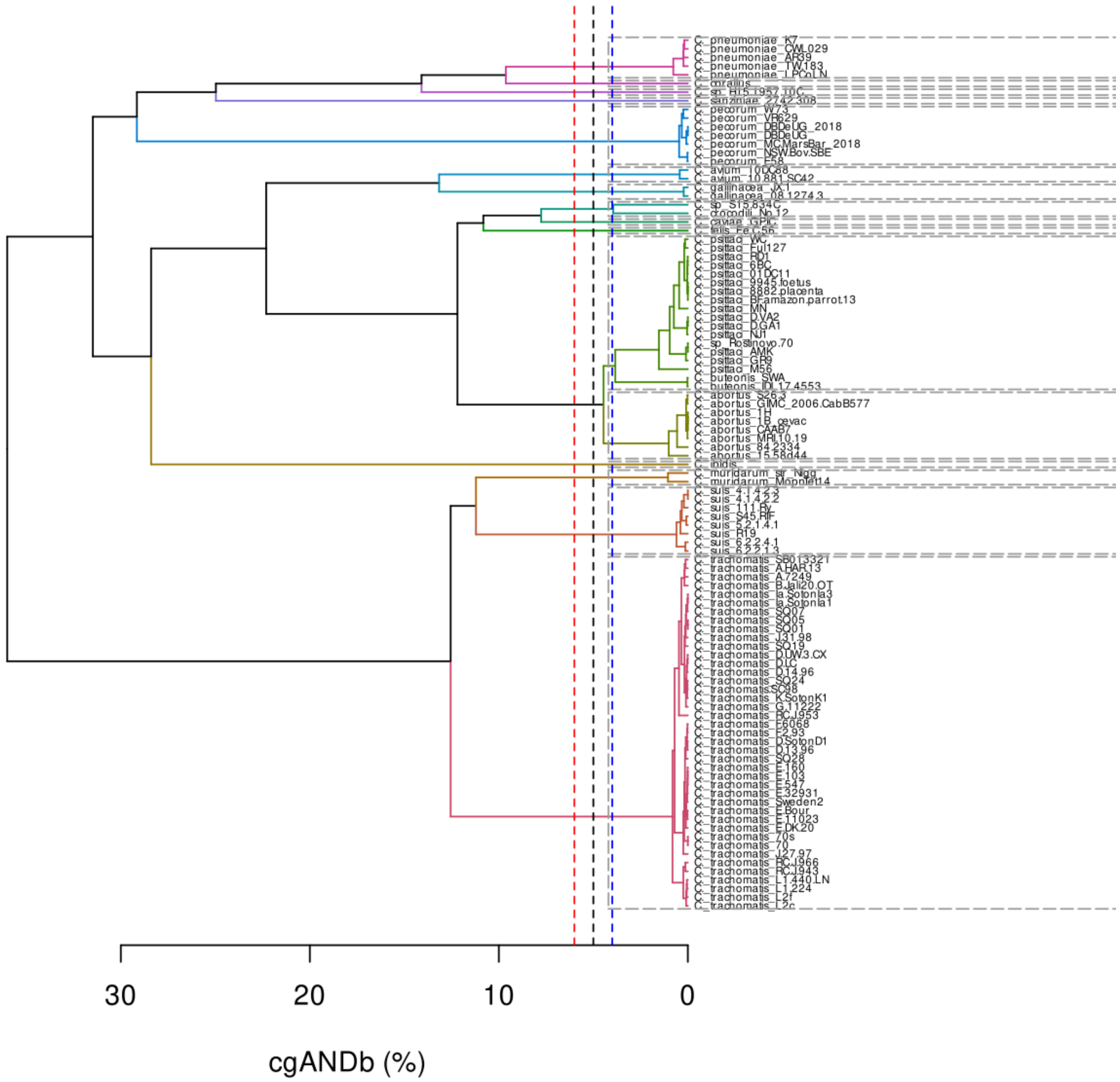

Figure S4. Application of an unsupervised learning approach to the core genome ANIb distance (cgANIb) matrix to identify statistically-consistent species-like clusters. The cgANIb matrix was converted to a distance matrix and clustered using the Ward.D2 algorithm. The optimal number of clusters (*k*) was determined with the average silhouette-width statistic.
